# Modeling the trend of coronavirus disease 2019 and restoration of operational capability of metropolitan medical service in China: a machine learning and mathematical model-based analysis

**DOI:** 10.1186/s41256-020-00145-4

**Published:** 2020-05-06

**Authors:** Zeye Liu, Shuai Huang, Wenlong Lu, Zhanhao Su, Xin Yin, Huiying Liang, Hao Zhang

**Affiliations:** 1 State Key Laboratory of Cardiovascular Disease, Fuwai Hospital, National Center for Cardiovascular Diseases, Chinese Academy of Medical Sciences and Peking Union Medical College, Beijing, 100037 China; 2 Clinical Data Center, Guangzhou Women and Children’s Medical Center, Guangzhou, 510623 Guangdong China; 3 School of Software & Microelectronics, Peking University, Beijing, 102600 China; 4 Heart center and Shanghai Institute of Pediatric Congenital Heart Disease, Shanghai Children’s Medical Center, National Children’s Medical Center, Shanghai Jiaotong University School of Medicine, Shanghai, 200127 China

## Abstract

**Background:**

To contain the outbreak of coronavirus disease 2019 (COVID-19) in China, many unprecedented intervention measures are adopted by the government. However, these measures may interfere in the normal medical service. We sought to model the trend of COVID-19 and estimate the restoration of operational capability of metropolitan medical service in China.

**Methods:**

Real-time data of COVID-19 and population mobility data were extracted from open sources. SEIR (Susceptible, Exposed, Infectious, Recovered) and neural network models (NNs) were built to model disease trends in Wuhan, Beijing, Shanghai and Guangzhou. Combined with public transportation data, Autoregressive Integrated Moving Average (ARIMA) model was used to estimate the accumulated demands for nonlocal hospitalization during the epidemic period in Beijing, Shanghai and Guangzhou.

**Results:**

The number of infected people and deaths would increase by 45% and 567% respectively, given that the government only has implemented traffic control in Wuhan without additional medical professionals. The epidemic of Wuhan (measured by cumulative confirmed cases) was predicted to reach turning point at the end of March and end in later April, 2020. The outbreak in Beijing, Shanghai and Guangzhou was predicted to end at the end of March and the medical service could be fully back to normal in middle of April. During the epidemic, the number of nonlocal inpatient hospitalizations decreased by 69.86%, 57.41% and 66.85% in Beijing, Shanghai and Guangzhou respectively. After the end of epidemic, medical centers located in these metropolises may face 58,799 (95% CI 48926–67,232) additional hospitalization needs in the first month.

**Conclusion:**

The COVID-19 epidemic in China has been effectively contained and medical service across the country is expected to return to normal in April. However, the huge unmet medical needs for other diseases could result in massive migration of patients and their families, bringing tremendous challenges for medical service in major metropolis and disease control for the potential asymptomatic virus carrier.

## Introduction

The outbreak of coronavirus disease 2019 (COVID-19) has been presenting a major threat to public health. The first COVID-19 case was reported on Dec 8, 2019 [1]. To curb the spread of the virus, Chinese health authorities have taken the strictest massive anti-epidemic actions since Jan 2020, including mass isolation, social distancing and community containment [2, 3]. Moreover, the government has implemented traffic restrictions across the whole country with massive reduction in public transportation capacity. As the epidemic situation remains fraught in China, key epidemiological questions, such as the effectiveness of implemented strategies for disease control, remain to be fully investigated.

The government has been increasingly investing medical resources in the treatment of patients with COVID-19. On February 5, three cabin hospitals and two other makeshift hospitals successively started to treat infected patients. By March 9, 346 medical teams and 42,600 medical professionals have been dispatched from other provinces across the country to combat the epidemic in Hubei province. It is reported that 7512 designated hospitals and related fever clinic are mobilized nationwide [4]. However, the nationwide mobilization of medical resources could severely disturb local routine medical service. According to the *2018 National Report on the Services, Quality and Safety in Medical Care System* [5], currently about 2 million patients each year travel across regions to seek medical care in China, among which 43% of all the cross-regional cases are concentrated in Beijing, Shanghai and Guangzhou (842 thousand cases in total). These three metropolises play a pivotal role in the healthcare system in China, providing high-quality medical service for patients in China. Notably, over thousands of medical professionals from medical centers in these metropolises have been dispatched to Wuhan and other cities in Hubei province to fight COVID-19 [6]. As the full resumption of normal healthcare services in the metropolises marks the complete restoration of healthcare system in China from the epidemic of COVID-19, providing estimation on the number of affected patients and prediction of restoration of routine medical service is urgently needed to facilitate preparedness of the healthcare system.

In this study, we provided an estimation of the epidemic trend of COVID-19 in Wuhan and representative metropolises in China and forecast the time point when the routine medical service would recover from the epidemic. Furthermore, we utilized data on population migration to construct an improved mathematical model to measure the impact of traffic restrictions on the migrant patients, providing estimation of operational pressure for metropolitan medical service after the end of the epidemic.

## Methods

### Data sources

In this study, data from two sources were used for statistical analysis. The website of Tencent news provided us with the time series data of COVID-19 by locations, including the number of confirmed cases, deaths, recovered cases, and newly diagnosed cases [7]. Baidu migration [8] is an open-source big data project visualizing population migration. Leveraging its Location based services system and Baidu Tianyan system, we obtained the daily migration scale index (MSI) of Beijing, Shanghai and Guangzhou, in January and February of both 2019 and 2020. The data involved in this study are available and public, provided by the media or common data platform. There is no need for approval from ethics committee as no privacy issue exists.

### Model construction and data analysis

#### Construction of a modified SEIR model

**Fig. 1:**
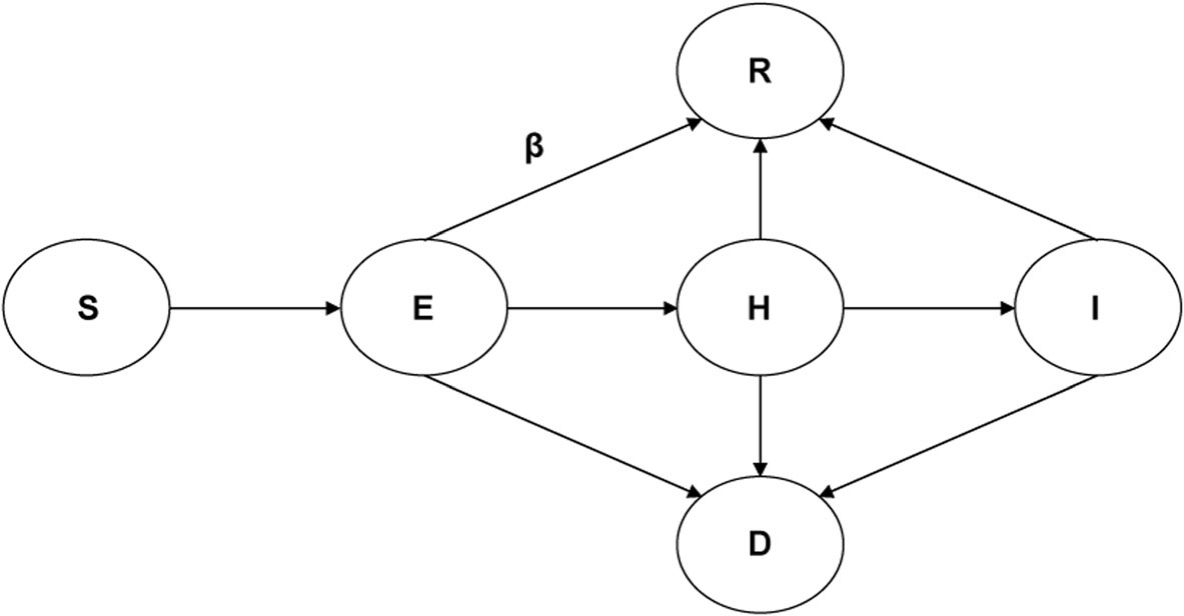
Susceptible, Exposed, Infectious, Recovered (SEIR) model diagram

In view of the actual situation of self-healing of the exposed people in this epidemic, we added the rehabilitation coefficient (β) based on the classic SEIR model [9] (Susceptible, Exposed, Infectious and Removed model) and in order to verify the effectiveness of the implemented interventions for epidemic control, we added the quarantine measures variable (in day 46) and the Cabin hospital variable (in day 59). In addition, a new House quarantine module (H) was added to demonstrate the effectiveness of new initiatives, as shown in Fig. 1 (See details in the Supplemental Materials Part 1). In order to estimate the epidemic situation of large medical centers and predict the time to restore the functions of daily medical service, we utilized data from Shanghai, Beijing, and Guangzhou to simulate the development of the epidemic assuming that only human-to-human transmission exists, no special medicine is found at this stage and no major health events happen [10–12]. In the meantime, special consideration was given to the epidemic situation in Wuhan, as it’s the epicenter of epidemic. We constructed a specific model to estimate the impact of community containment and construction of makeshift hospitals on the epidemic situation in Wuhan. The date when the first confirmed case was detected in local government was used as the first day for modeling the local epidemic situation, specifically, Dec 8, 2019 in Wuhan [1], Jan 13, Jan 12 and Jan 21, 2020 respectively in Beijing, Shanghai and Guangzhou [13–15].Furthermore, we adopted a strategy different from that of the previous cumulant-based modeling methods [9]. We counted the daily number of people excluding dead and recovered cases to ensure the independence of the current status of each patient, so as to guarantee the accuracy and interpretability of the SEIR model.

#### Construction of time series prediction model based on neural network

The purpose of the neural network (NNs) is to supplement overcome the limitations of the SEIR model [16]. On the basis of the prediction of inflection point by the SEIR model, we used the neural network to further refine the fitting at specific time points to achieve accurate prediction. In order to obtain the optimal neural network model, we chose four network structures that was commonly used to predict the cumulative number of the confirmed cases nationwide, as shown in Enclosure. According to R^2^ (R-squared/coefficient of determination) and the loss function, the model with the best performance is selected to further predict other epidemic changes, including the number of suspects, cures, and deaths, where the R^2^ is defined as and is the predictive value of our model.

The predicted results are visualized using Tensorflow tools for further analysis. (See the Supplemental Materials Part 2).

#### Construction of inference model for inpatient hospitalization based on ARIMA

In order to quantify the impact of the outbreak on migrant patients during the persecuting period of epidemic, we utilized the Baidu migration big data platform to extract migration data for 1 month before and after the Spring Festival of Beijing, Shanghai and Guangzhou in 2019 and 2020. Using the Baidu Migration Index [7] as a measure of the number of immigrants and the Chinese lunar calendar date as a standard, in terms of daily units, we construct a curve of the reduction in the number of migrants in 2020 compared to 2019.

Autoregressive Integrated Moving Average model (ARIMA) is used to predict the date when the amount of migration can return to a normal status [17]. Considering that February 10th is the presumed date of returning to work issued by the Chinese government, and 8th and 9th are amid the peak period of the return trip, we only use data after the 10th for the prediction. The ARIMA model contains three hyper-parameters: Auto-Regressive, Integrated, and Moving Average. Based on the estimated declining proportion of metropolitan immigration population, and the number of cross-regional cases in Beijing, Shanghai and Guangzhou in 2019, we estimated the declining proportion of patients whose demands for cross-regional medical service are suppressed during the outbreak. The features of the autocorrelation function and partial autocorrelation function were utilized to debug the model parameters, and the R2 was used as a final evaluation standard. (See the Supplemental Materials Part 3).

#### Statistical analysis

The Discrete variables including the daily number of confirmed cases, deaths and recovered cases were collected for modeling based on machine learning. The recovered rate and mortality were calculated to fit the SEIR model. The estimated number of patients with unmet medical services was indicated with median, and statistical uncertainty was presented using 95% confidence interval (CI). Python3.5 (Python Software Foundation) and Statistical Product and Service Solutions (SPSS21.0, Almonk, New York, USA) were used here for data analysis.

## Result

**Fig. 2:**
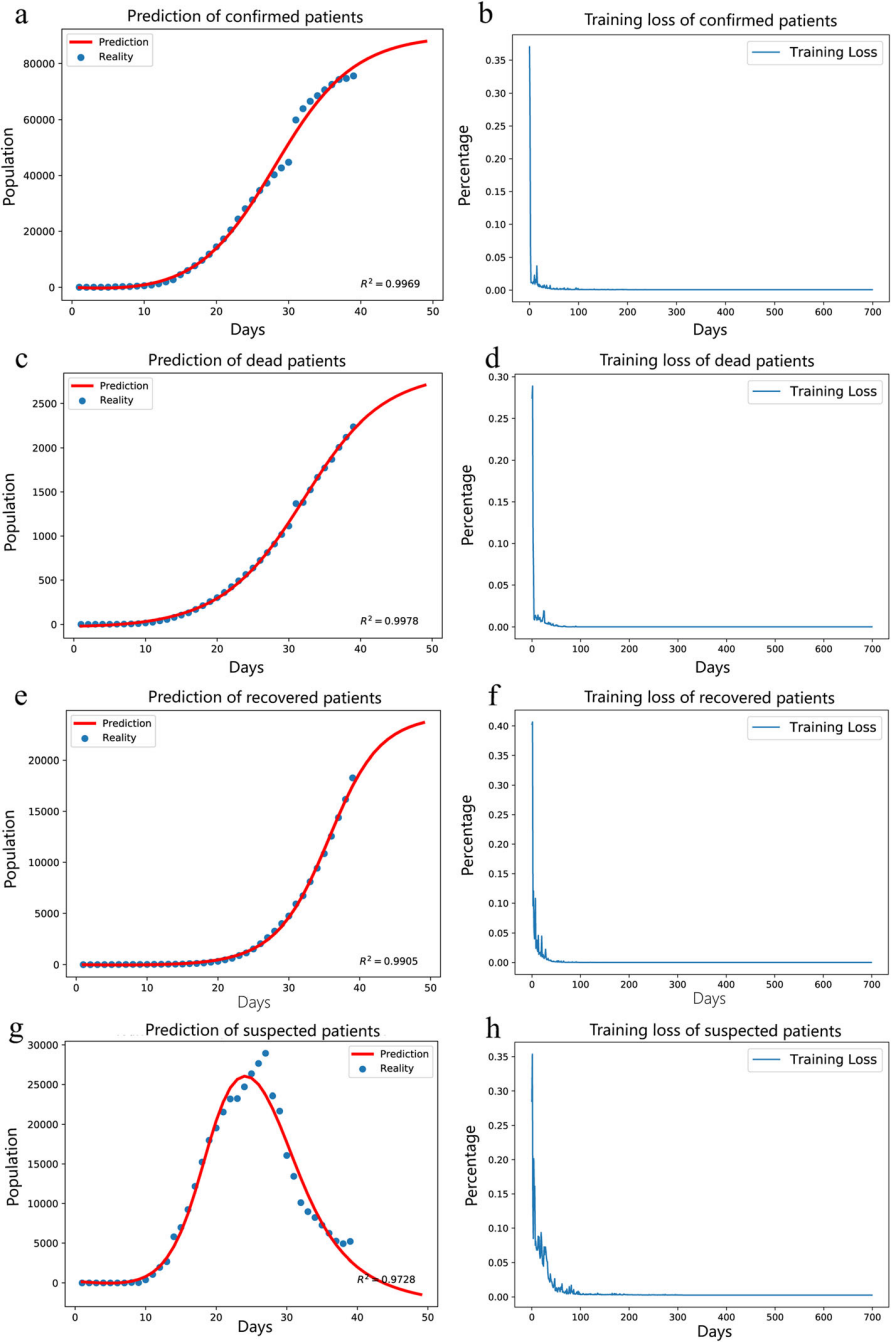
The estimation of the number of confirmed patients, dead patients, recovered patients and suspected patients based on multilayer perceptron. **a**, **c**, **e**, **g** The fitting effect on the number of confirmed patients, dead patients, recovered patients and suspected patients in China. **b**, **d**, **f**, **h** The loss curves fitting the number of confirmed patients, dead patients, recovered patients and suspected patients in China

According to our model-based analysis, the estimated number of infected patients nationwide would reach to the peak of 80,000 during the persecuting period of the epidemic (Fig. 2). The short-term trend described by the neural network was consistent with the modified SEIR model. Therefore, the modified SEIR model was further used to predict long-term trends of the epidemic. The modified SEIR model simulated the consequences of several measures, including quarantine, the increment of medical professionals and beds. Wuhan was locked down 46 days after the outbreak of the epidemic, and in the meantime other cities strictly restricted the movement of population and initiated home-based quarantine. As a result, the daily growth of exposed people was significantly controlled and gradually dropped down to 15,000 people, which fundamentally minimize the epidemic risk of COVID-19.

**Fig. 3:**
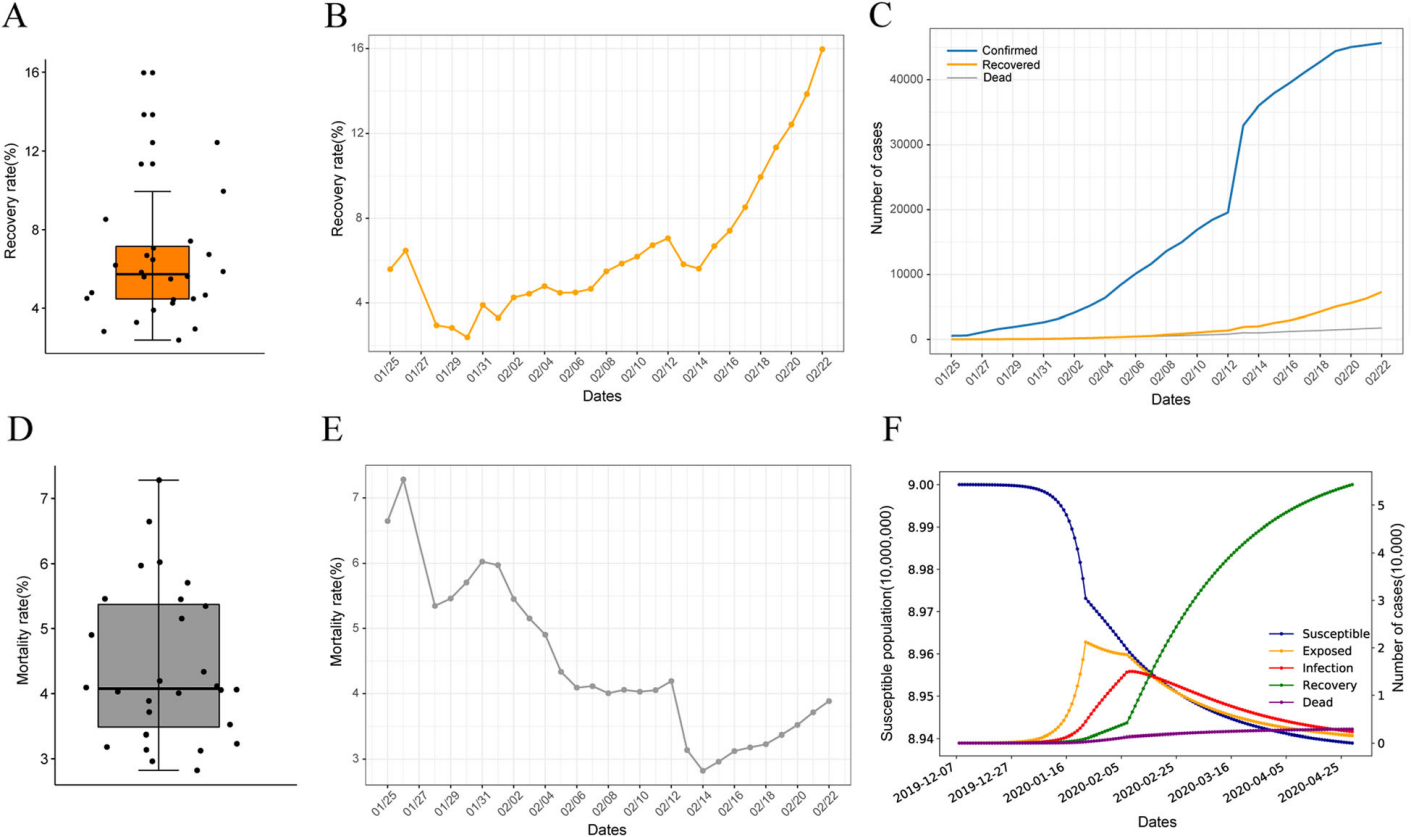
SEIR model of Wuhan and its dependence graph. **a, b** Box chart and line chart of recovery rate in Wuhan. **c** Line chart of the number of confirmed cases, recovered cases and dead cases in Wuhan. **d**, **e** Box chart and line chart of mortality rate in Wuhan. **f** SEIR model estimates the trend of epidemic situation in Wuhan

**Fig. 4:**
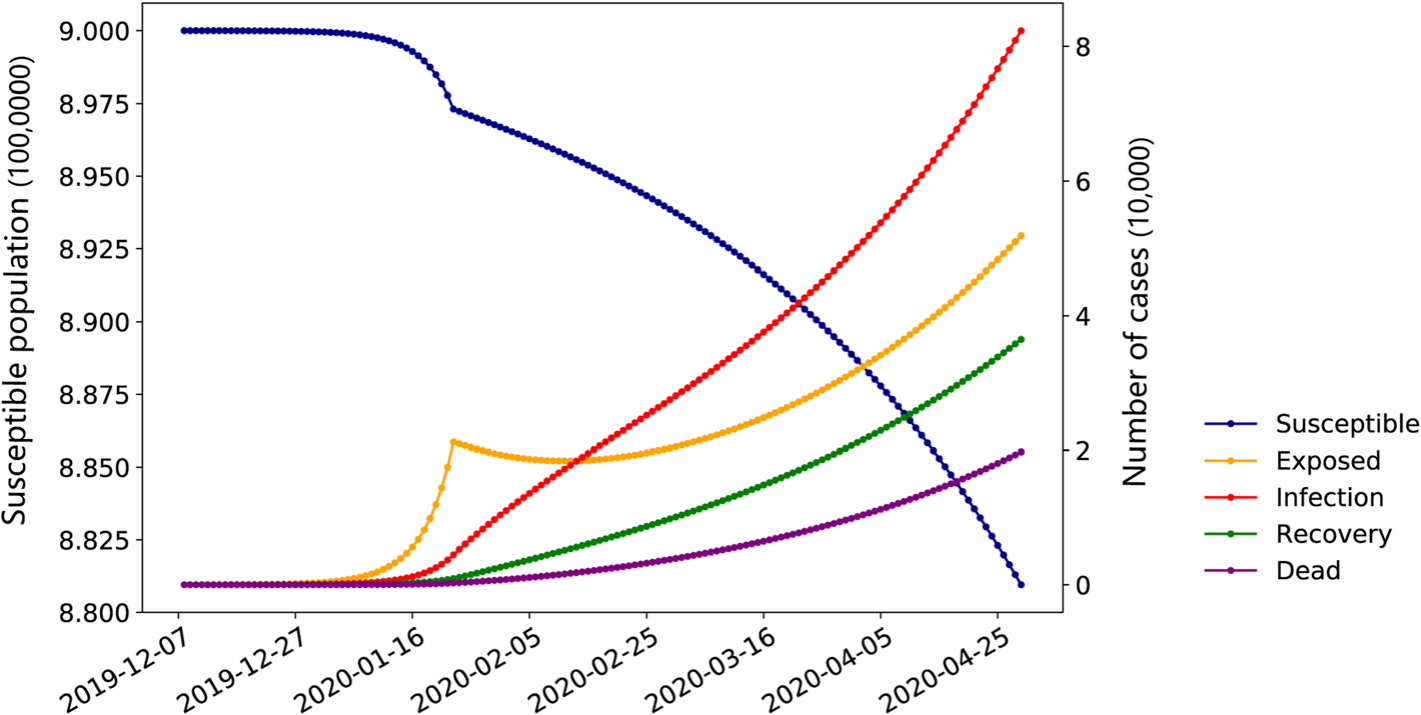
SEIR model simulates the epidemic situation in Wuhan without additional beds

According to our analysis, with the timely mobilizing of medical professionals and the expansion of beds capacity, the number of infected cases decreased significantly while the number of recovered cases increased significantly. The recovery rate increased by more than 10% and the death rate decreased by 2.5%. The peak of the epidemic occurred earlier than expected. The modeling results show that if the government simply conducted city-wide quarantine in Wuhan without additional beds and reinforced medical professionals, the total number of infected cases might reach more than 80,000 and the number of deaths might be around 20,000, which increases by 45% and 567% respectively. The peak of the epidemic in Wuhan might be postponed beyond April (Figs. 3 and 4).

**Fig. 5:**
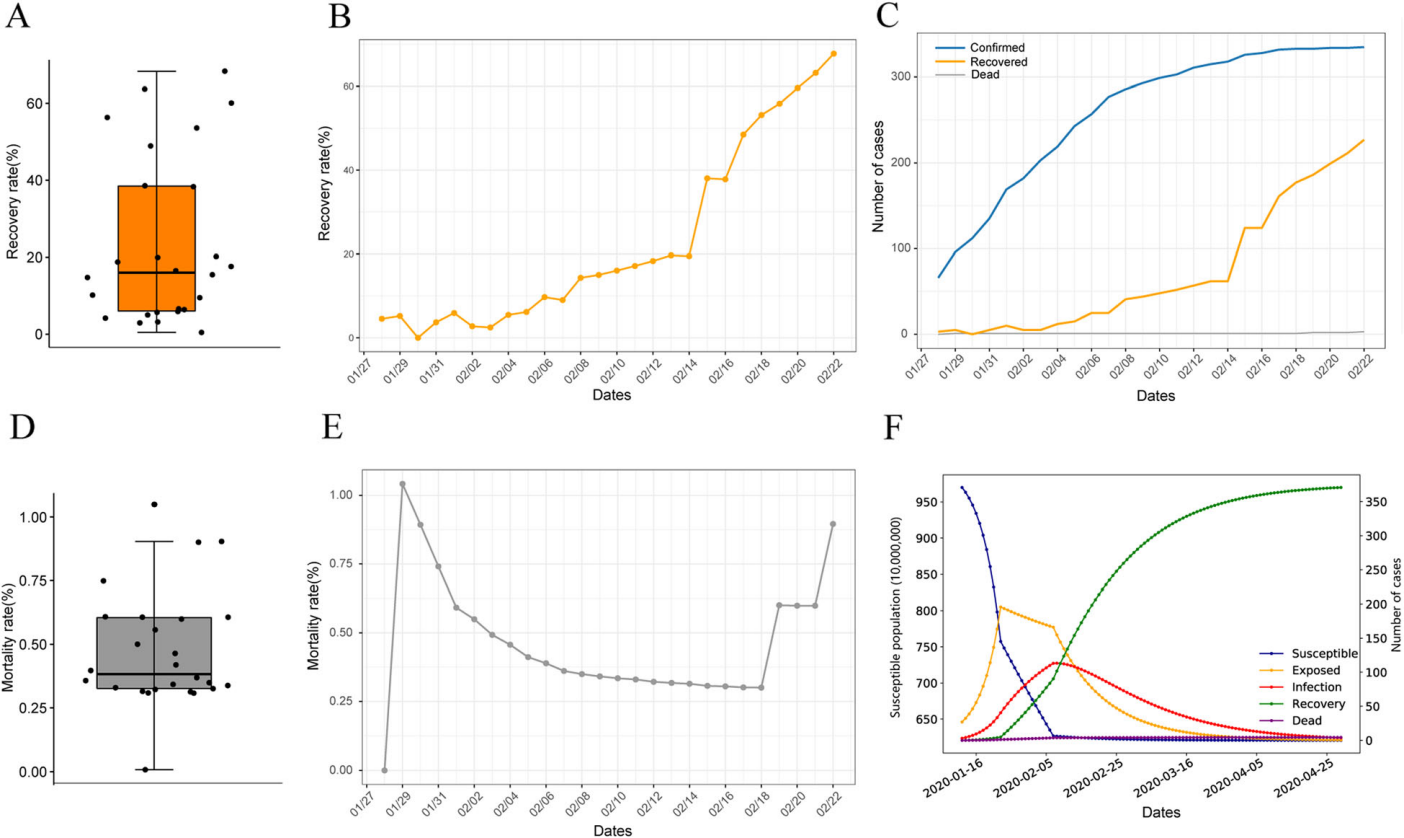
SEIR model of Shanghai and its dependence graph. **a, b** Box chart and line chart of recovery rate in Shanghai. **c** Line chart of the number of confirmed cases, recovered cases and dead cases in Shanghai. **d**, **e** Box chart and line chart of mortality rate in Shanghai. **f** SEIR model simulates the trend of epidemic situation in Shanghai

**Fig. 6:**
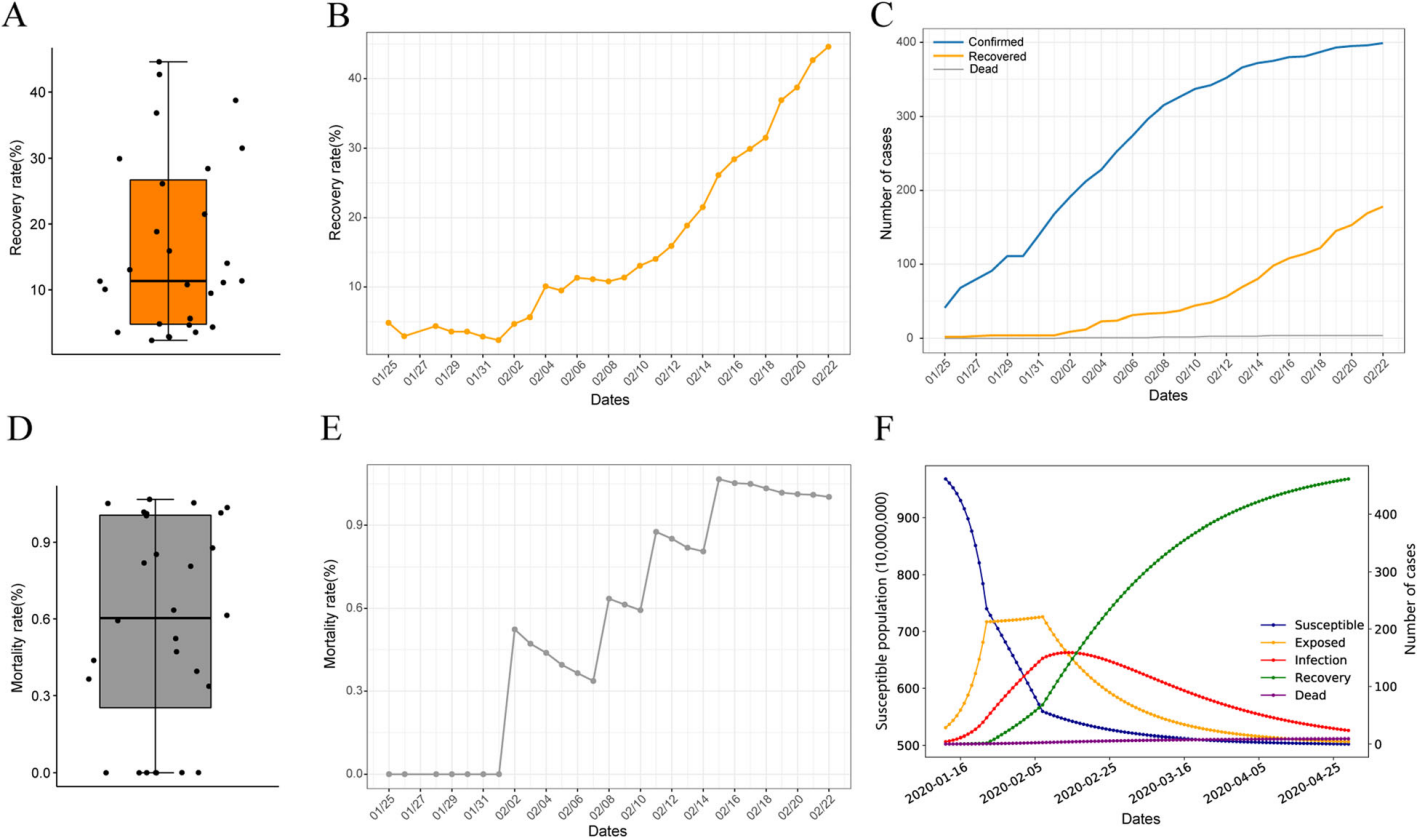
SEIR model of Beijing and its dependence graph. **a**, **b** Box chart and line chart of recovery rate in Beijing. **c** Line chart of the number of confirmed cases, recovered cases and dead cases in Beijing. **d**, **e** Box chart and line chart of mortality rate in Beijing. **f** SEIR model simulates the trend of epidemic situation in Beijing

**Fig. 7:**
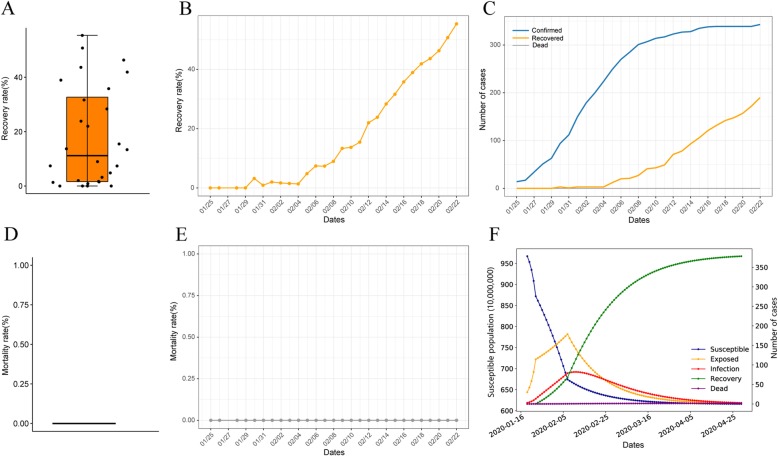
SEIR model of Guangzhou and its dependence graph. **a**, **b** Box chart and line chart of recovery rate in Guangzhou. **c** Line chart of the number of confirmed cases, recovered cases and dead cases in Guangzhou. **d**, **e** Box chart and line chart of mortality rate in Guangzhou. (F) SEIR model simulates the trend of epidemic situation in Guangzhou.

The implementation of traffic restrictions led to better containment of COVID-19 infection in other parts of China. The recovery rate in Beijing, Shanghai and Guangzhou rose steadily to more than 45%, with a death rate lower than 1%. According to the estimation, there will be 480 confirmed cases in Beijing, 360 in Shanghai, and 380 in Guangzhou. Nevertheless, the death rates in Beijing, Shanghai and Guangzhou are significantly lower than that of Wuhan (1% vs. 4%), while the recovery rates are comparatively higher (45% vs. 16%), suggesting the dispatch of medical professionals have limited negative impact on the local epidemic control. This finding suggests that the growth of epidemic has been slowing down and is gradually reaching the peak. Based on the deduction results of MLP model, the epidemic of Wuhan may reach its inflection point at the end of this March and come to an end in April. The outbreak in Shanghai (Fig. 5), Beijing (Fig. 6) and Guangzhou (Fig. 7) will end about 1 month earlier than expected. Given that all medical professionals engaged in the epidemic treatment should be quarantine for 2 weeks, the national healthcare system (except Wuhan) would return to normal in mid-April.

**Fig. 8:**
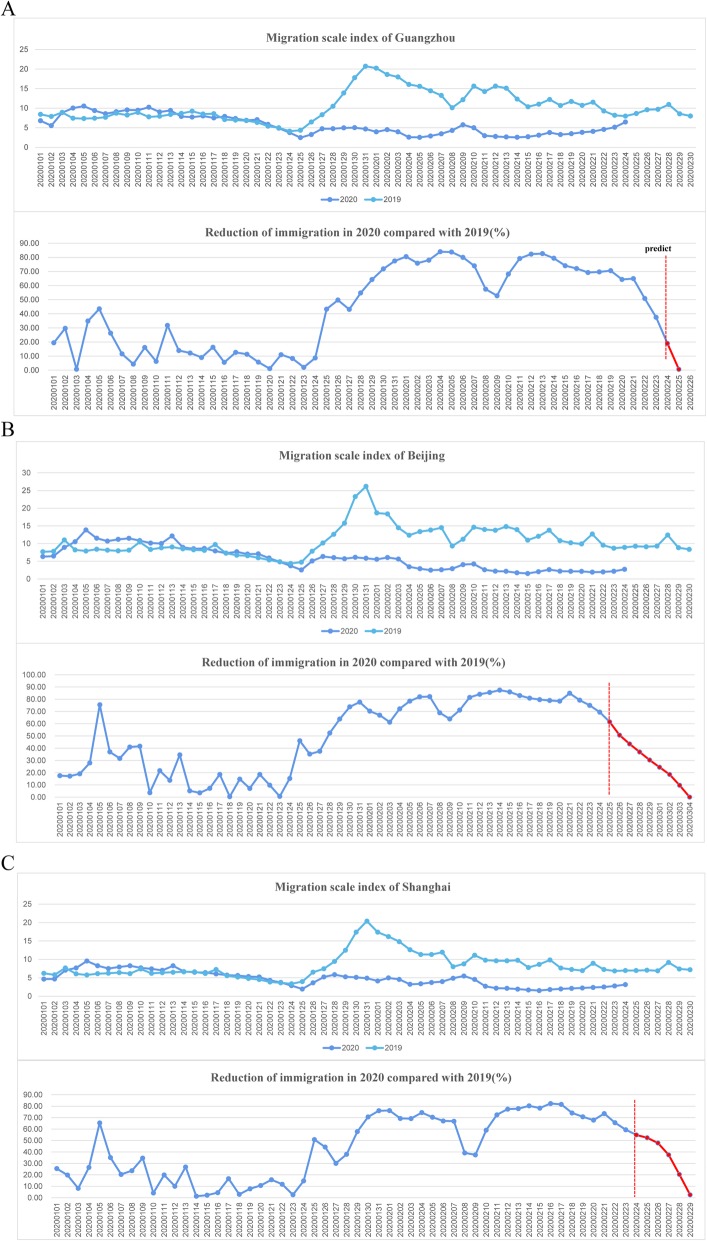
The migrant population of three metropolises in the first quarter of 2019 and 2020. **a** Line chart of migration scale index and reduction of immigration in 2020 compared with 2019(%) of Beijing. **b** Line chart of migration scale index and reduction of immigration in 2020 compared with 2019(%) of Guangzhou. **c** Line chart of migration scale index and reduction of immigration in 2020 compared with 2019(%) of Shanghai.

The migrant population of Beijing, Shanghai and Guangzhou in the first quarter of 2019 and 2020 was shown in Fig. 8. The medical centers from three metropolises may face more than 58,799(95%CI 48926–627,232) additional hospitalizations in total in the first month after the epidemic. The estimated number of patients with unmet medical service (hospitalization) during the epidemic in Beijing, Shanghai and Guangzhou was shown in Table 1.

**Table 1:** Summarized estimation of epidemic situation in Wuhan, Beijing, Shanghai and Guangzhou

	Wuhan	Shanghai	Beijing	Guangzhou
**The estimated end time of the epidemic**	April 20th–30th	March 16th–30th	March 16th–30th	March 16th–30th
**The estimated inflection point of the epidemic**	March 12th–19th	March 2rd-9th	March 2rd-9th	March 2rd-9th
**The estimated number of cumulative confirmed cases**	55,000	360	460	380
**The estimated number of patients with unmet medical service (hospitalization) during the epidemic (95%CI)**	\	23,279 (17,967-27,504)	25,264 (22,151-28,364)	10,256 (8808-11,364)

## Discussion

The ongoing outbreak of COVID-19 is a major public health emergency, posing great challenges to the public healthcare system [18, 19]. As Chinese authorities have taken massive anti-epidemic actions, the spread of the virus has been effectively curbed. However, patients’ demand for routine medical care is substantially suppressed during the persecuting period of the epidemic, especially for patients with noninfectious chronic diseases. In this study, we constructed a mathematical model to simulate the epidemic situation in Wuhan and other parts in China (represented by Beijing, Shanghai and Guangzhou). Compared to Wu’s study [20], we conducted multi-model analysis and prediction, with special consideration given to the massive anti-epidemic actions taken by Chinese authorities, such as community containment, large-scale mobilization of medical professionals and rapid expansion of beds capacity. In line with Chen’s study, which points to the effectiveness of massive interventions taken by China on the control of COVID-19 epidemic [21], we estimated that the end of COVID-19 would occur about 1 month earlier, ending in late April in Hubei and late March in the rest region of China (except Hubei). The recovery of national medical service was estimated to be observed in mid-April. However, the rapid expansion of migrant patients early after the end of the epidemic would bring significant pressure to medical service in Beijing, Shanghai and Guangzhou.

The distribution of medical resources in China is relatively imbalanced [5]. For instance, 66% of all the established National Clinical Research Centers are located in Beijing, Shanghai and Guangzhou, and there are significant numbers of patients across the country admitted in these institutions each year [22]. The restoration of operational capability of metropolitan medical centers is determined by several factors, including the epidemic control in critically infected region (represented by Hubei province), the regional epidemic control, and the recovery of public transportation and population flows nationwide [5]. In the present study, we utilized the network data to model the trend of epidemic containment in Wuhan and those three metropolises, and conducted a model-based analysis to estimate challenges after the restoration of operational capability.

The SEIR model is a classic epidemiological model widely used for modeling infectious diseases with incubation period [9]. In this study, we adapted and modified the classic SEIR model based on the characteristics of COVID-19, such as the viral transmission capability, its spatial distribution and route of transmission. We introduced the House quarantine module (H), the rehabilitation coefficient (β), the quarantine measures variable and the cabin hospital variable. Moreover, the real-time network data was leveraged in this modified SEIR model. The traditional SEIR model is flawed by inaccuracy to predict the inflection point of the epidemic [23]. We used neural network to address this issue. Compared to the traditional time series prediction algorithms, the neural networks process the distributed parallel information by coordinating the interconnection among massive internal nodes [24]. Hence, it is superior in large-scale parallel processing, distributed storage, elastic topology, high redundancy and high robustness, making it more suitable for high-speed non-linear operations. As such, we combined the neural networks with SEIR model, constructing model based on official data to compare the prediction accuracy among four commonly used neural networks and adopted the optimal model for the prediction for the epidemiological trend of COVID-19.

As the epidemic expands, the confirmed cases are continuously up-rising. However, according to the government report, except for Hubei province, the total number of newly confirmed cases in mainland China has declined for 16 consecutive days [25], indicating the epidemiological situation of COVID-19 is in a slow-growth (except for Hubei province), while the growth rate of infected cases in Hubei is still in a steady increase, which is also corresponding to our model-based prediction.

Amid the severe epidemic, patients’ demands for routine healthcare in other specialties are typically suppressed, especially for cross-regional cases. Admittedly, telemedicine among large medical centers may partially relieve the pressure on patients’ demand for medical service, whereas the treatment for major diseases, especially invasive surgical treatment, is less available for patients. These treatments, in most cases, cannot be provided by local community medical institutions.

The implementation of traffic restrictions is one of the main causes hindering large medical centers from returning to normal order of medical service. As public transportation is being shut down, a large number of patients are having difficulty to access large medical centers, leading to a waste of medical resources among these medical centers. To visualize the impact of this issue, we utilized the Baidu Migration Platform to obtain definitive data of population migration index among Beijing, Shanghai and Guangzhou. Based on the ARIMA model, we estimated that the number of cross-regional patients in major medical centers among those three large cities would be reduced by approximately 59 thousand during the first quarter of 2020. The predictions based on this model might be reliable because of its advantage in forecasting time series when compared to the general mathematical models like regression analysis and linear function. According to our analysis, an extra 59 thousand migrant patients would head to the major medical centers in Beijing, Shanghai and Guangzhou, posing a great challenge to the metropolitan healthcare system. Thus, Chinese health authorities should make adequate preparation for triaging patients and their accompanied families. In addition, sufficient attention must be paid to the infected patients with excessively long incubation period and asymptomatic 2019-nCoV carriers [26]. Since 70% of patients are cross-regional cases in tertiary hospitals in Beijing, massive number of migrant patients would raise challenges to local epidemic control during the early stage after the containment of COVID-19. The relevant authorities should develop strategies for health inspection and quarantine. Our results also underscore the importance of a hierarchical healthcare system, which relies on regional medical centers to triage patients.

Our study had several limitations. Firstly, the SEIR model-based analysis was conducted based on open source data, it could not fully capture the actual number of infected cases, and the imported confirmed cases from overseas were not included in our model-based analysis. Secondly, the predictive model was constructed based on the natural distribution of people, it cannot be applied to the special population distribution such as welfare institute. Thirdly, this model is unable to accurately predict the epidemiological trend of COVID-19 under the cases of viral mutation and the development of specific anti-virus therapy. Fourthly, the increment of medical professionals involved and beds capacity followed an un-uniform growth pattern, which cannot be simulated by our models. Lastly, the psychological factors may cause a bias to our predictive models, as patients’ intention to seek medical care would be reduced under the shadow of the epidemic.

## Conclusions

In conclusion, our study highlights the significant challenges presented to the healthcare system in China under a public health emergency. As the resolute massive anti-epidemic actions are implemented, the end of the outbreak would be expected in late March in mainland China outside Hubei province and the routine medical service would recover in mid-April. However, patients’ demand for routine medical care would expand rapidly within a month after the end of outbreak highlighting the need of coordination among regional medical centers. These findings could inform policy makers and public health officials to devise preparedness plans to address the unmet medical needs of other diseases under the COVID-19 epidemic.

## Supplementary information

**Additional file 1.**

## Data Availability

All the primary data and materials involved in this paper are from the published articles and web links, and they are all available online.

## Abbreviations

COVID-19: Coronavirus disease 2019
SEIR: Susceptible, exposed, infectious and recovered
NNs: Neural network models
ARIMA: Autoregressive integrated moving average
MSI: Migration scale index
H: House quarantine module
MLP: Multilayer perceptron model
ICU: Intensive care unit
RNN: Recurrent neural network
LSTM: Long short-term memory
GRU: Gate recurrent unit
