# Supplementary material for: Modeling the trend of coronavirus disease 2019 and restoration of operational capability of metropolitan medical service in China: a machine learning and mathematical model-based analysis
Source: Glob Health Res Policy. 2020 May 6;5:20. doi: 10.1186/s41256-020-00145-4 (PMC7200323; doi:10.1186/s41256-020-00145-4)
Supplement: Supplementary file 1 — Additional file 1. [file 41256_2020_145_MOESM1_ESM.doc]

**Supplemental Materials**

**Part 1**

An modified SEIR model was used in this study to estimate the epidemic trend of COVID-19. Presuming the target population is an isolated population, which would shrink exclusively because of viral infection. Since the infected patients are contagious during incubation period, and there is possibility of self-healing, we introduced a self-healing coefficient and a home isolation module to parameterize the actual situation. In addition, given that the recovery rate and the mortality of infected patients would be affected by several interference factors, such as the impaired operation capacity of hospitals, the impact of measures taken by authorities, and the occurrence of the vaccine, we modified the relevant parameters of the model. The model stratified the population into S, E, I, R, D, and H. The viral transmission can be characterized by the differential equations of these six categories, as shown below:


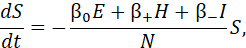


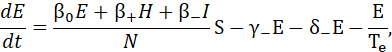


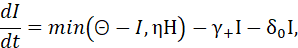


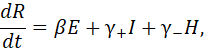


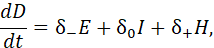


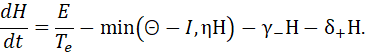


The meaning of the relevant parameters are shown in the following Supplemental table 1:

| Parameter | Description | Parameter | Description |
| --- | --- | --- | --- |
| 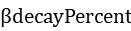 | The declining proportion of infectious rate after the implementation of quarantine | 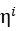 | Patients’ intention for hospitalization when the outbreak emerged |
| 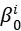 | The probability of susceptible population being infected by incubatory carrier | β | The self-healing rate of exposed population |
| 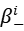 | The probability of susceptible population being infected by quarantined inpatients | 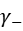 | The cured rate of home-based quarantined individuals |
| 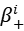 | The probability of susceptible population being infected by home based quarantined individuals | 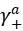 | The cured rate of quarantined inpatients after the measures are strengthened |
| 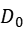 | The death toll at time zero | 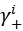 | The cured rate of hospitalized infected patients |
| 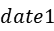 | The date when measures are implemented | 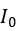 | The number of hospitalized infected patients at time zero |
| 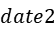 | The date when measures are strengthen | 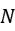 | Total population |
| 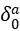 | The mortality of quarantined infected patients after the implementation of epidemic control measures | 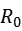 | The cured cases at time zero |
| 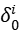 | The mortality of quarantined infected patients | 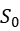 | Susceptible population at time zero |
| 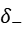 | The mortality of incubatory carrier | 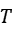 | The total time of the viral transmission |
| 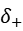 | The mortality of home based quarantined individuals | 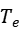 | The incubation period of the disease |
| 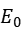 | The exposed population at time zero | 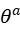 | Beds capacity after the epidemic control measures are strengthen |
| 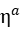 | Patients intention for hospitalization after the epidemic control measures are strengthen | 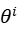 | Beds capacity at time zero |

**Supplemental table 1: The parameters of our SEIR model**

**Part 2**

The time series of epidemic is a kind of non-linear and non-stationary data with multiple noises, which can be affected by uncertain factors including anthropogenic and external intervention, whereas possess has a very strong non-linear fitting ability. It can automatically extract the characteristics of time series and constantly adjust the super parameters to obtain better results through the setting of training set and test set. In this study, four NNs architectures were used to predict the epidemic situation, including MLP, RNN and its extension algorithm: LSTM and GRU，as is shown in Supplemental figure 1.

|  | **network structure** |
| --- | --- |
| **Recurrent Neural Network (RNN)** | 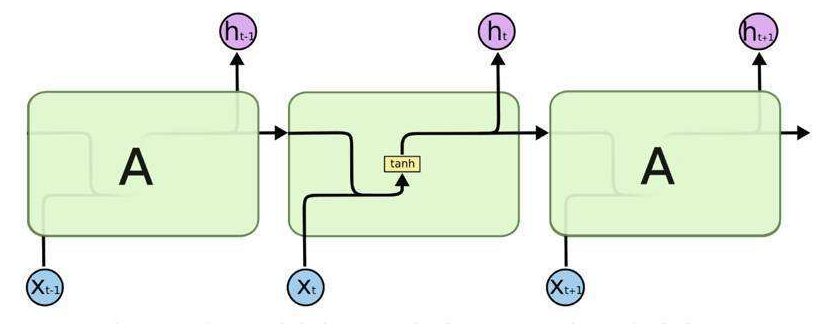 |
| **Long Short-Term Memory (LSTM)** | 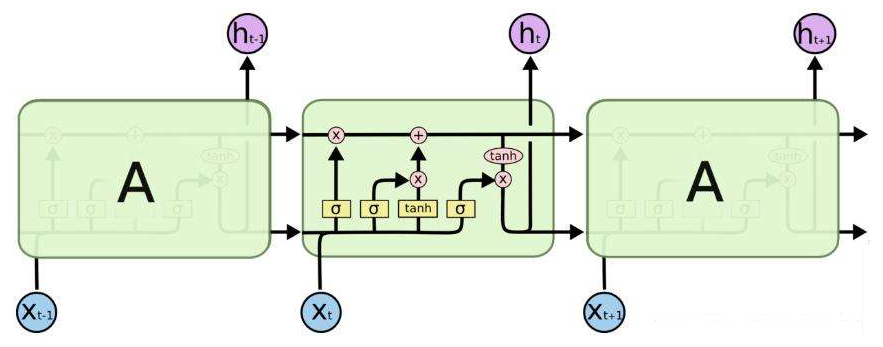 |
| **Gate Recurrent Unit (GRU)** | 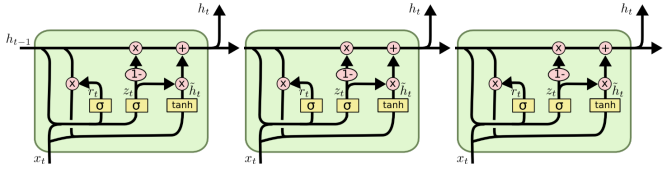 |
| **Multilayer Perceptron (MLP)** | 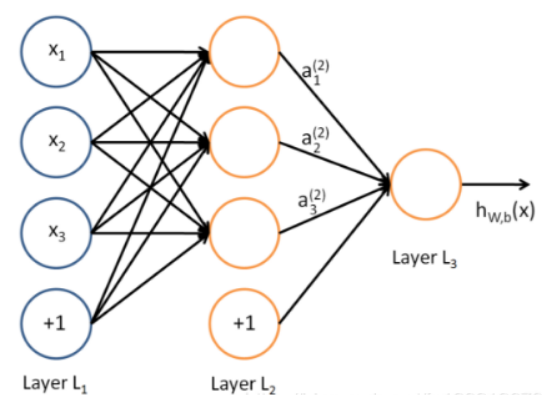 |

**Supplemental figure 1：A summary of four neural network architectures**

MLP is also known as DNN, which can be divided into three parts: input layer
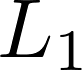
, hidden layer
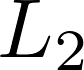
, and output layer
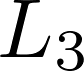
 according to the position of different layers. Notably, the number of the hidden layer ,noted as
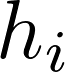
,maybe more than one. Each layer is composed of one or more neurons. The sum of the number of hidden layers and output layers is recorded as the depth
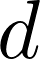
 of the network, and each layer corresponds to an activation function
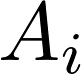
. Then there will be some formulas as follow:


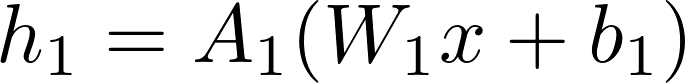


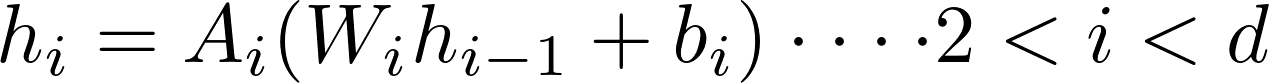


Where
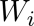
 is the weighted value of each connection, and
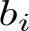
 is a partial value. The common activation functions include S-type function, linear piecewise function, and
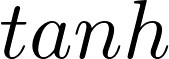
 function. The reason why we choose
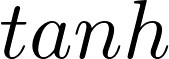
 as the activation function here is that its input and output can maintain a nonlinear monotonic rising and falling relationship, which meets the gradient conditions of the neural network. Apart from this,
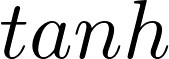
 has good fault tolerance and is bounded. The expression is as follows:


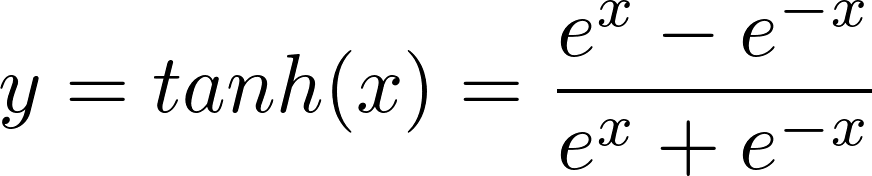


The most prominent feature of RNN is that the output of the neuron at a certain time can be input to the neuron again. This tandem structure is very suitable for time series data, because it can maintain the dependency in the data. Repeated structures can be obtained for the expanded RNN and the parameters in the network are shared, which greatly reduces the neural network parameters that need to be trained. However, a large number of practices have shown that standard RNNs are often difficult to achieve long-term storage of information, and there are problems of gradient disappearance and gradient explosion. Therefore, LSTM and GRU have added an internal gating mechanism to maintain long-term dependencies. Compared with the hidden units of RNNs, their internal structure is more complicated, and with the flow of information along the network, they can be selectively added or reduced by adding linear intervention. The propagation mechanism can be identified as follows:


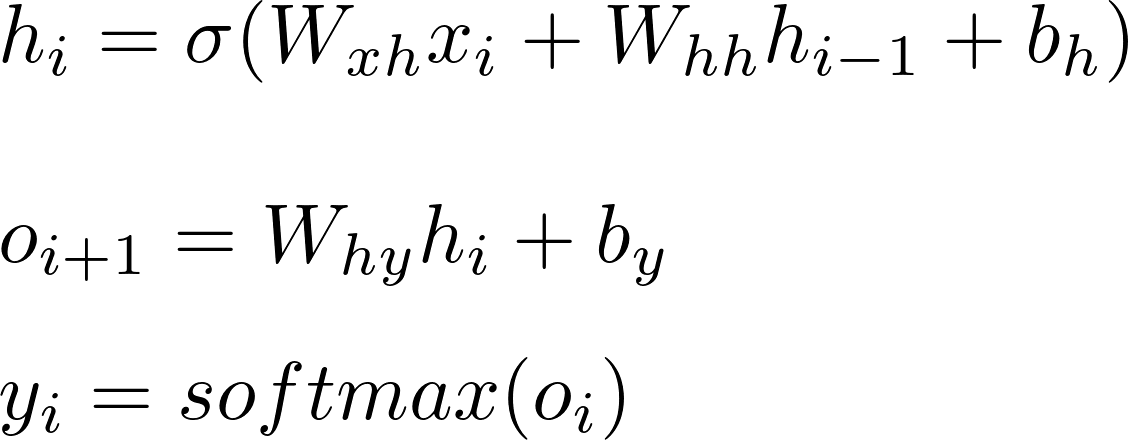


Where
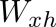
 is the weight matrix of input unit to hidden unit;
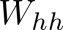
 is the connection weight matrix of hidden unit;
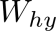
 is the connection weight matrix of hidden unit to output unit;
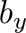
 and
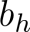
 are offset vectors. All four models are implemented by R language.

We utilized four kinds of neural network to estimate the number of infected cases nationwide，the optimal one was shown in the manuscript, and the other three structures are shown in Supplemental figure 2.

| 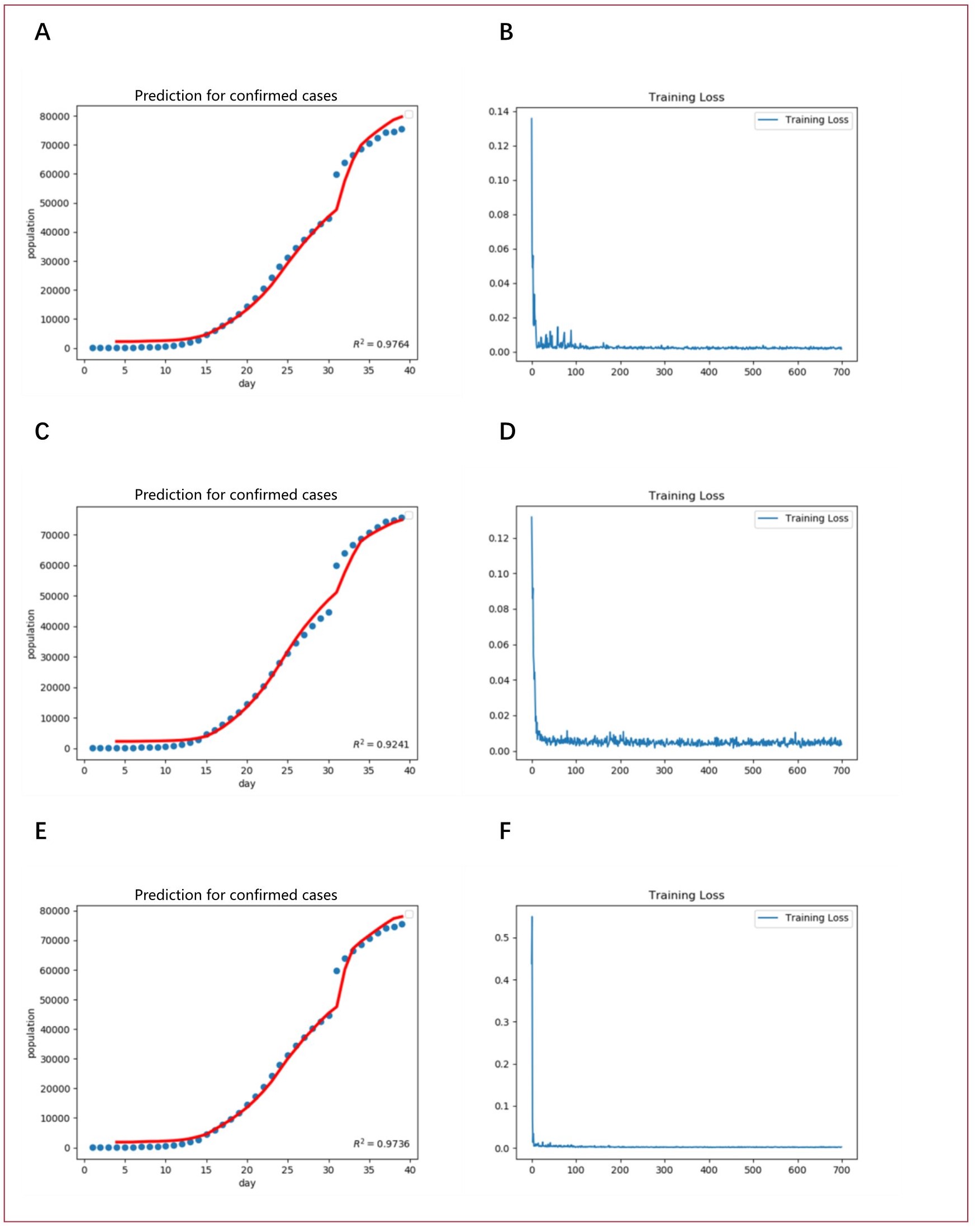 |
| --- |
| **Supplemental figure 2*: Use cyclic networks to fit the confirmed number of people in China***  (A)(C)(E) The fitting effect of GRU (Gated Recurrent Unit), LSTM (Long Short Memory Network) and RNN (Recurrent Neural Network) models respectively. (B)(D)(F) The loss curves of GRU, LSTM and RNN models respectively. |

**Part 3**

The ARIMA (p, d, q) model was used to estimate the urban migrant population in this study. It includes three parameters, the Auto-Regressive term p, the Moving Average term q, and the Integrated term d. It has a better performance on the stable short-term time series or stable time series after the difference operation. Due to the independence of the data before and after February 10th,which is the date of return to work stipulated by Chinese government , we choose the time period after it for predictive analysis. Considering the length of the time series, ARIMA model is a better choice. In order to obtain the optimal model, after converting the original sequence to a stationary sequence through a first-order difference operation, we can approximate the parameter values of p and q by using the trailing and truncating features of the autocorrelation function and the partial autocorrelation function, as shown in Supplemental figure 3. During the fitting process using the SPSS tool, the parameter value is continuously adjusted by using R^2^ as the standard. The fitted parameters of the final output of Beijing, Shanghai, and Guangzhou are ARIMA (4,1,1) _(R2 = 0.826)_, ARIMA (3,1,1) _(R2 = 0.799)_, and ARIMA (3, 1,1) _(R2 = 0.801)_,showing the potential for accurate assessment of follow-up medical needs.


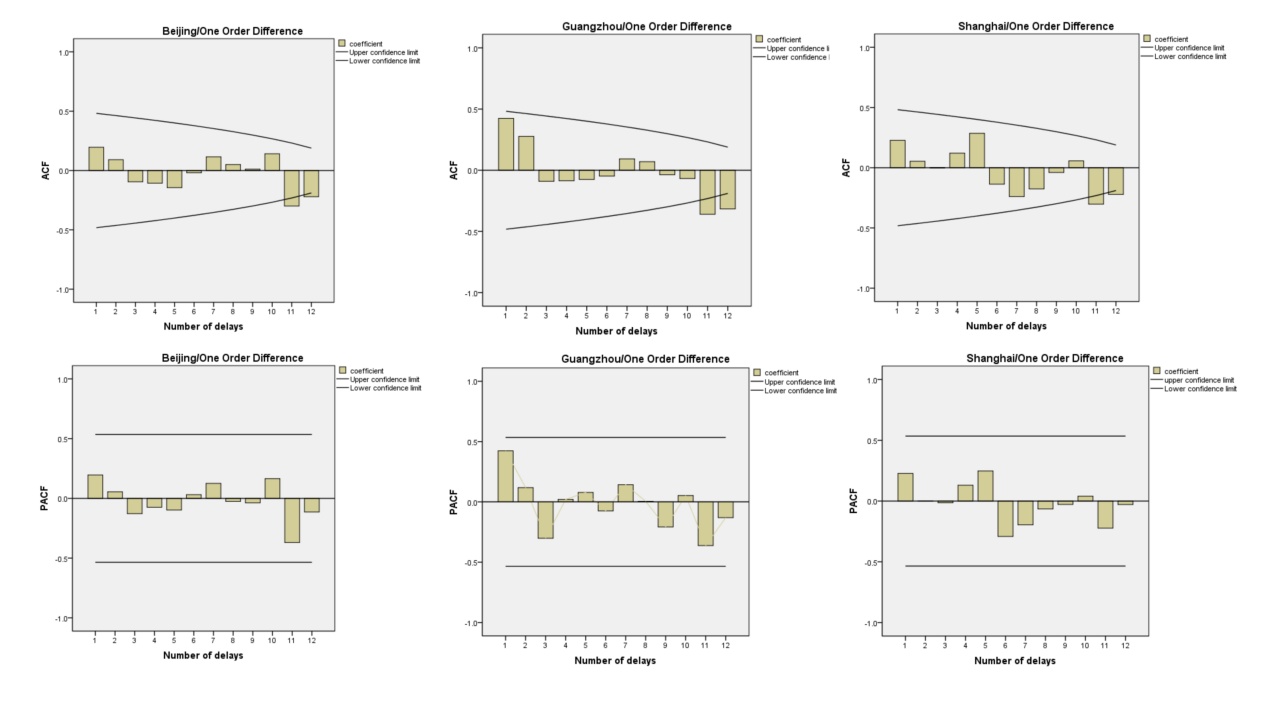


**Supplemental figure 3: The ACF and PACF of the data after the first-order difference operation**
